# It is time for reform: Results from a questionnaire survey on the current status of next generation HBP surgeons in Japan

**DOI:** 10.1002/jhbp.12092

**Published:** 2024-12-10

**Authors:** Yukiko Kosai‐Fujimoto, Tomoaki Yoh, Takanobu Hara, Saori Umezawa, Aya Maekawa, Yasuko Matsuo, Norihiro Ishii, Hiroko Okinaga, Itaru Endo, Masayuki Ohtsuka, Susumu Eguchi, Ken Shirabe

**Affiliations:** ^1^ Next Generation Project Working Group in Japanese Society of Hepato‐Biliary‐Pancreatic Surgery Tokyo Japan; ^2^ Department of Surgery NHO Fukuokahigashi Medical Center Fukuoka Japan; ^3^ Department of Surgery and Science, Graduate School of Medical Sciences Kyushu University Fukuoka Japan; ^4^ Department of Surgery, Graduate School of Medicine Kyoto University Kyoto Japan; ^5^ Department of Surgery Nagasaki University Graduate School of Biomedical Sciences Nagasaki Japan; ^6^ Department of Gastroenterological and General Surgery St. Marianna University School of Medicine Kanagawa Japan; ^7^ Division of Hepatobiliary and Pancreatic Surgery, Cancer Institute Hospital Japanese Foundation for Cancer Research Tokyo Japan; ^8^ Department of Hepatobiliary and Pancreatic Surgery, Graduate School of Medicine Institute of Science Tokyo Tokyo Japan; ^9^ Department of Surgery Nara Medical University Nara Japan; ^10^ Division of Hepatobiliary and Pancreatic Surgery, Department of General Surgical Science, Graduate School of Medicine Gunma University Gunma Japan; ^11^ Department of Surgery Gunma Saiseikai Maebashi Hospital Maebashi Gunma Japan; ^12^ Department of Hepato‐Biliary‐Pancreatic Surgery Tokyo Metropolitan Cancer and Infectious Diseases Center Komagome Hospital Tokyo Japan; ^13^ Japanese Society of Hepato‐Biliary‐Pancreatic Surgery Tokyo Japan; ^14^ Department of Gastroenterological Surgery Yokohama City University School of Medicine, Graduate School of Medicine Yokohama Japan; ^15^ Department of General Surgery Graduate School of Medicine, Chiba University Chiba Japan

**Keywords:** career building, questionnaire survey, work‐life balance, young surgeons

## Abstract

A questionnaire survey was designed and performed to assess the current status of “next generation” hepatobiliary and pancreatic (HBP) surgeons regarding surgical training, career, recruiting, and work‐life balance in Japan. Using a valid email address, a questionnaire was sent to members of the Japanese Society of Hepato‐Biliary‐Pancreatic Surgeons (JSHBPS) who were under 45 years old. The questionnaire comprised 50 questions across the following four sections: (i) board certification of JSHBPS, (ii) research activity and overseas study, (iii) recruiting, and (iv) work‐life balance. A questionnaire survey was sent to 1735 HBP surgeons and responses were received from 303 members (17.5%). In a survey with 303 respondents, over 45.9% were above 41 years old, 93.7% were male, and 91.0% were affiliated with university surgery departments. About 25.1% were JSHBPS board‐certified, while 72.7% of uncertified doctors aspired for the certification. Research activity was deemed crucial by 74.9%. Recruitment targeting postgraduate years 1–5 was recommended, with the technical difficulty of surgery being the main reason for choosing HBP. Regarding work‐life balance, excessive work and classical work style were regarded as a hindrance to the sustainability of working practices. This survey highlighted that next generation HBP surgeons are highly motivated to acquire advanced surgical skills and recognize the importance of research experience. However, they are facing long working hours and insufficient training opportunities. Fundamental reforms, such as revising the training curriculum, improving work styles, and enhancing recruitment, are necessary steps forward to ensure the sustainability of HBP surgery in Japan.

## Graphical abstract

On behalf of the Next Generation Project Committee of the Japanese Society of Hepato‐Biliary‐Pancreatic Surgery, Kosai‐Fujimoto and colleagues conducted a questionnaire survey of society members under 45. The results highlight harsh work conditions, stressing the need for urgent improvements to ensure the sustainability of hepatobiliary and pancreatic surgery in Japan.

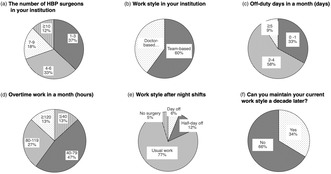

## 1 INTRODUCTION

Nowadays, the landscape for surgical professionals in Japan is in a transitional phase, triggered by a decreasing number of surgeons, especially in gastrointestinal surgery,^1^ and the implementation of legislation concerning work‐style reforms (Figure S1).^2^ Among the subspecialties in gastrointestinal surgery, hepatobiliary and pancreatic (HBP) surgery demands not only advanced knowledge but also exceptional surgical skills,^3^, ^4^, ^5^, ^6^, ^7^ outlining the challenging and time‐consuming road to professionalism; however, it should be noted that the field of HBP surgery is not exempt from this transitional phase.

Recognizing the need to align HBP surgery practices with this contemporary trend, the Japanese Society of Hepato‐Biliary‐Pancreatic Surgery (JSHBPS) took a gradual step by developing a dedicated working group named the Next Generation Project (NGP). Comprising JHBPS members aged 45 years or younger and supervised by a senior professor, the NGP working group formally initiated its activities by conducting a questionnaire survey to confirm the needs and current circumstances of the “next generation” in HBP surgery.

In this report, we present the results of the questionnaire survey. Clarifying the needs and current circumstances of the next generation HBP surgeons allows for developing the fundamental policy of the NGP working group, furthering the society's commitment to fostering a progressive and sustainable HBP surgery.

## 2 METHODS

### 2.1 Questionnaire survey

The questionnaire was sent to members of the JSHBPS who were 45 years old or younger, using a valid email address. Additionally, to minimize the influence of nonresponses, we configured the survey platform to require responses for all key variables, allowing only surveys with no missing data to be submitted. The questionnaire was developed based on consensus within the NGP working group and supervisor, comprising the following four categories: (i) JSHBPS board certification, (ii) research activity and overseas study, (iii) recruiting, and (iv) work‐life balance. The complete content of this questionnaire is detailed in the supplementary document—Data S1. The questionnaire survey was sent out just once to minimize duplication.

### 2.2 Data analysis

In this survey, various questioning styles were employed. The majority of questions offered only a single answer choice, and these were converted into percentages for graphical representation if appropriate. For questions with multiple answer choices, the data were presented using the actual numbers and corresponding groups. In selected questions, percentages or numbers were divided according to specific groups. The survey primarily consisted of “closed questions”, and the results of this survey consisted of descriptive statistics. Additionally, the data highlighted in the Results section 3 were determined in agreement by NGP members.

## 3 RESULTS

The questionnaire survey was sent to 1735 JSHBPS members on December 24, 2021, and the responses were obtained from 303 members (17.5%) until January 31, 2022.

### 3.1 Study population

The study population is shown in Table 1. Out of the 303 respondents, 139 (45.9%) were over 41 years old, followed by 107 (35.3%) aged 36 to 40, 49 (16.2%) aged 31 to 35, and eight (2.6%) aged 24 to 30. Similarly, the distribution of postgraduate years (PGY) among respondents reflected comparable proportions. The vast majority of respondents consisted of male HBP surgeons, accounting for 93.7% (*n* = 284), and were affiliated with university or academic centers (*n* = 276, 91.1%). At present, nearly half of the respondents (*n* = 156, 51.5%) worked in university hospitals or university‐associated hospitals. Among them, 81.7% (*n* = 247) were regularly employed as staff doctors, and 12.3% (*n* = 37) were Ph.D. students (Supplementary document section 1—Data S1).

**TABLE 1:** Study population in the questionnaire survey.

Age	Years old	24–30	31–35		36–40		41–45	
	*N* (%)	8 (2.6%)	49 (16.2%)		107 (35.3%)		139 (45.9%)	
PGY	Years	3–5	6–8		9–11	12–17	≥ 18	
	*N* (%)	7 (2.3%)	26 (8.6%)		38 (12.5%)	164 (54.1%)	68 (21.4%)	
Sex		Male			Female			
	*N* (%)	284 (93.7%)			19 (6.3%)			
Affiliation to universities		Yes			Never/quit			
	*N* (%)	276 (91.0%)			27 (8.9%)			
Place of work		University/ university‐related			Public hospital	Private hospital		
	*N* (%)	156 (51.8%)			93 (30.9%)	52 (17.3%)		
Current certifications^a^		General surgery board	GI surgery board	HBP surgery board	Endoscopy surgery	Da Vinci operator	Ph.D.	None
	*N* (%)	293 (96.7%)	265 (87.5%)	76 (25.1%)	54 (17.8%)	26 (8.6%)	196 (64.7%)	10 (3.3%)
Most desired certification		HBP Surgery board	Endoscopic Surgery		Da Vinci Operator	Ph.D.	All acquired	
	*N* (%)	165 (54.5%)	76 (25.1%)		26 (8.6%)	24 (7.9%)	12 (4.0%)	

Abbreviations: GI; gastrointestinal, HBP; hepatobiliary pancreatic; PGY, post‐graduate year; Ph.D., doctor of philosophy.

^a^ The question with multiple answers.

#### 3.1.1 The board certification system of JSHBPS

**FIGURE 1:**
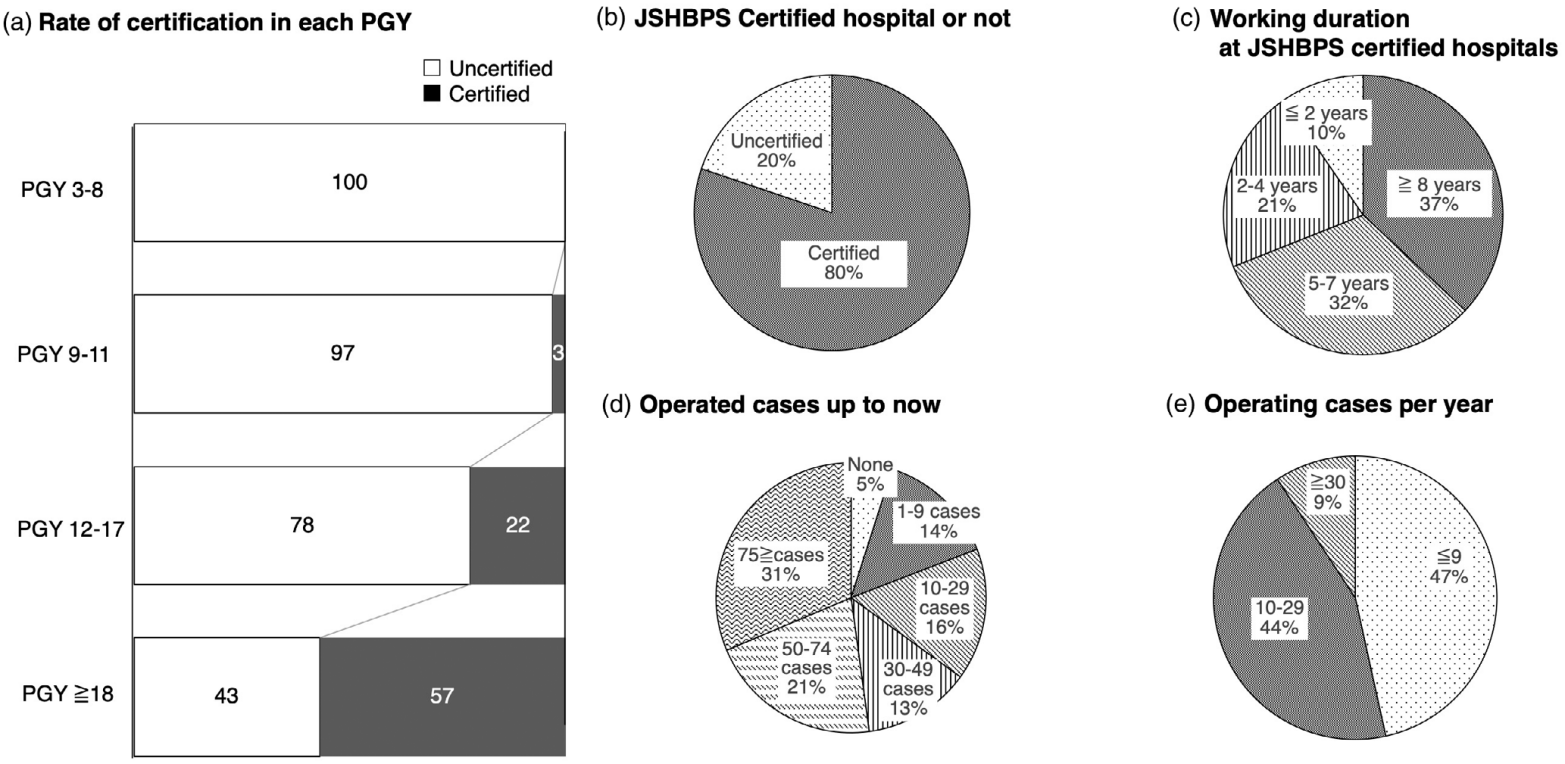
The board certificate of JSHBPS. (a) The current certification status according to the PGY. (b) The proportion of respondents working at board‐certified hospitals. (c) The duration of working at board‐certified hospitals. (d) The total number of highly advanced HBP surgeries that each respondent has experienced to date. (e) The number of highly advanced HBP surgeries performed by each respondent per year. JSHBPS, Japanese Society of Hepato‐Biliary‐Pancreatic Surgery; PGY, post‐graduate year; HBP, hepatobiliary pancreatic.

The section regarding the board certification of JSHBPS included six questions. The details of the board certification are described in Figure S2. As shown in Table 1, 25.1% of the respondents had already obtained JSHBPS board certification, and 72.7% of uncertified surgeons answered this board certification was their most prioritized one. Regarding the certification in each PGY, 57.4% of doctors in PGY 18 or older were already certified, 22.0% in PGY 12 to 17, and 2.6% in PGY 9 to 11 (Figure 1a). A total of 80% of all respondents worked in board‐certified hospitals, including 156 surgeons in university or university‐related hospitals (Figure 1b). Surgeons must have worked in board‐certified hospitals for more than 3 years in the last 7 years and have performed more than 50 highly advanced HBP surgeries to be eligible for applying for this certification. In this context, about two‐thirds of the surgeons (*n* = 209, 69.0%) had worked for more than 5 years in such hospitals, and the total number of operated cases so far was over 50 in 52.1% of the respondents (Figure 1c,d). The number of highly advanced surgeries operated in a year is shown in Figure 1e. The most common response was less than nine cases (*n* = 141, 46.5%) and 10–29 cases (*n* = 134, 44.2%).

The last question in this section was given to the respondents' free opinions about the board certification system. Respondents mainly focused on (i) clarifying the judge system, (ii) the loosening of applicant requirements, and (iii) providing financial incentives after certification (supplementary document section 2—Data S1).

#### 3.1.2 Research activity and overseas study

**FIGURE 2:**
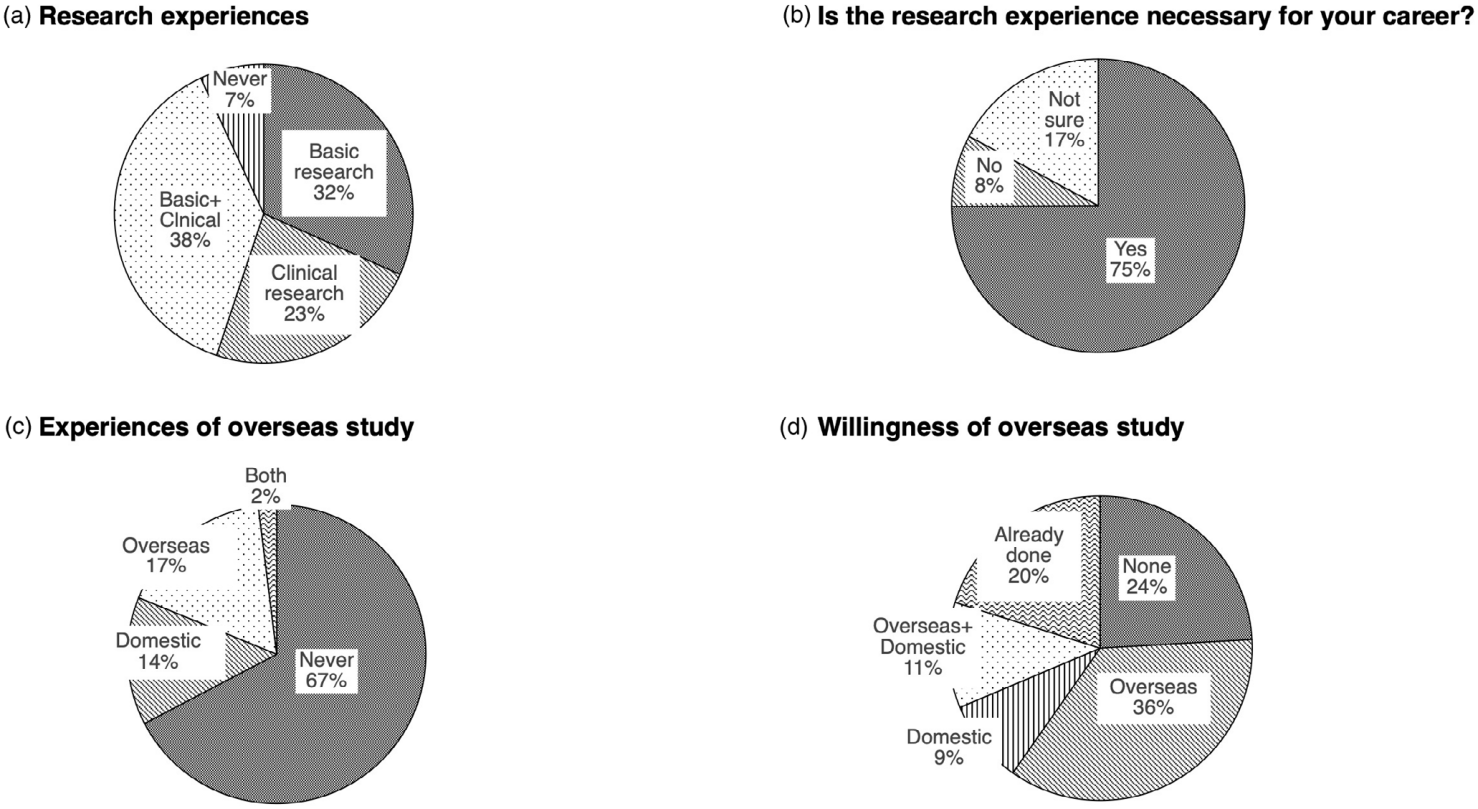
The research activity and overseas study. (a) The experience in clinical or basic research. (b) The responses to whether research experience is necessary for their career. (c) The experience or lack of experience in studying outside their affiliated institutions. (d) The responses to the question, “Do you want to study abroad?.”

The section regarding the research activity and overseas study included 11 questions. Most of the respondents had already experienced the clinical, basic, or both types of research, and three‐quarters of the respondents answered that the research activity was necessary for their career formation (Figure 2a,b). The detailed number of each respondent's published articles is shown in the supplementary document section 3—Data S1. Meanwhile, approximately 70% of respondents had never been to overseas or domestic institutions for research or training, although most of them expressed the willingness to do so (Figure 2c,d).

#### 3.1.3 Recruiting

**FIGURE 3:**
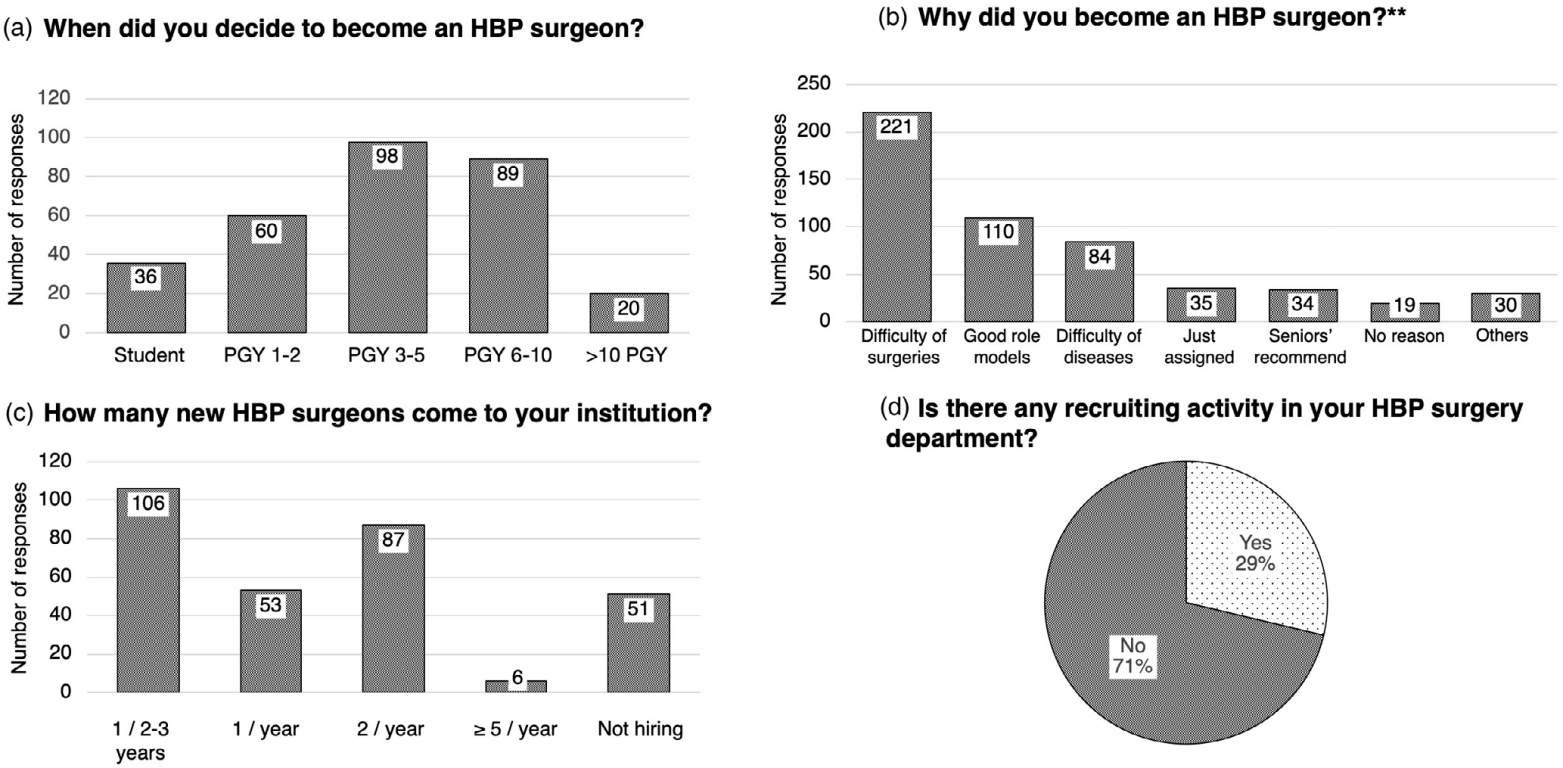
Recruitment activity. (a) The timing of when respondents decided to become HBP surgeons. (b) The respondents' reasons for becoming HBP surgeons. (c) The annual number of new surgeons entering their institution. (d) The proportion of respondents with or without recruitment activities. ** = questions with multiple answers. HBP, hepatobiliary pancreatic; PGY, post‐graduate year.

The section regarding recruiting included 11 questions. Recruiting HBP surgeons is one of the biggest concerns for surgeons who suffer from short‐staffed and burdening working environments of HBP surgeons in Japan. Among the respondents, 98 (32.3%) decided to specialize in HBP surgery during their PGY 3–5, 89 (29.4%) during PGY 6–10, 60 (19.8%) during PGY 1–2, and 36 (11.9%) had already aspired to be HBP surgeons even before graduating from medical school (Figure 3a). In the question of the reason for choosing HBP surgery, the most common reason was the technical complexity of surgeries (*n* = 221, 72.9%), followed by the influence of good role models (*n* = 110, 36.3%), and the variety of challenging diseases (*n* = 84, 27.7%) (Figure 3b). Correspondingly, the answer to the question “What is important in the recruitment of HBP surgeons?” was “Show how HBP surgeries are challenging, rewarding, and attractive.” (*n* = 238, 78.5%) (supplementary document section 4.4—Data S1). Regarding the number of newcomers in each institution, 106 respondents (35.0%) answered only ONE surgeon in a few years, while six respondents (2.0%) answered more than five doctors per year (Figure 3c). Meanwhile, regarding the recruitment activities, 87 surgeons answered yes (28.7%) with ideas like holding orientation seminars and social gatherings for young candidates, while 216 (71.3%) answered no due to lack of time, lack of human resources, or because they are already recruiting them to be general surgeons first (Figure 3d).

#### 3.1.4 Work‐life balance

**FIGURE 4:**
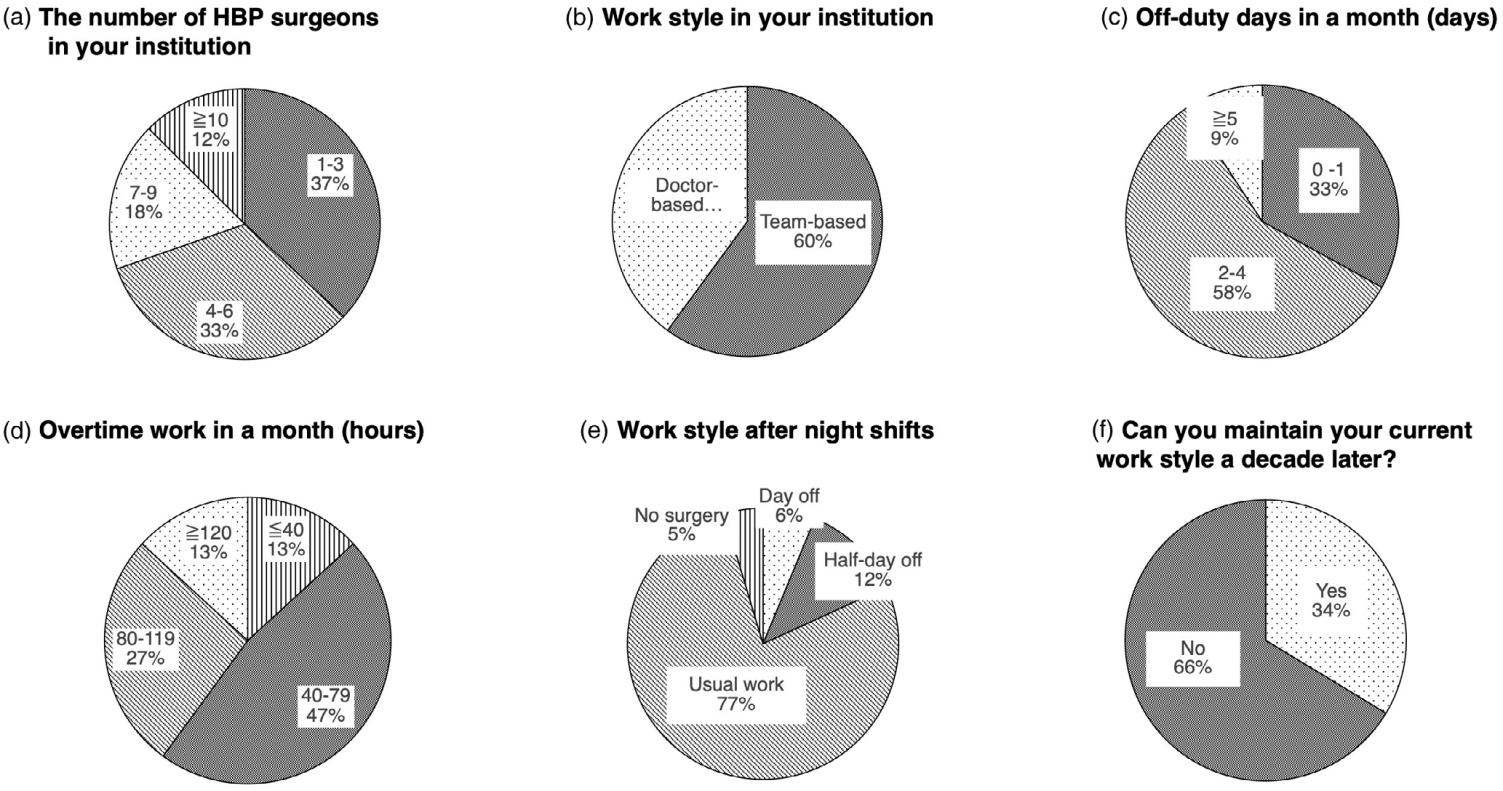
Work‐life balance. (a) The number of HBP surgeons in the institution. (b) The work style of the institution. (c) The number of off‐duty days per month. (d) The overtime working hours per month. (e) The work style after night shifts. (f) The responses to the question, “Do you think you can maintain your current work style a decade from now?”. HBP, hepatobiliary pancreatic.

The final section focused on the work‐life balance and working environment of HBP surgeons. This section included 22 questions. Figure 4a shows the distribution of HBP staff in the respondents' hospitals, with one‐third answering 1–3 staff(s), another one‐third answering 4–6 staff, and 12.5% answering more than 10 staff. Another chart regarding the number of female staff shows that 71.0% of respondents have no female colleagues, and only 1.7% had more than three female surgeons (supplementary document section 5.3—Data S1). In the question about the working style, 60.1% are working with a team‐based style, while 39.9% are with a surgeon‐based style (Figure 4b). Figure 4c–e feature the working environment, highlighting the scarcity of off‐duty days or short paid leave of surgeons. Additionally, overtime work exceeding 80 h was found in one‐third of them, despite the upcoming government policy that bans overtime work longer than 80 h. When asked whether they could maintain their current work style a decade later, two‐thirds answered “No” (Figure 4f).

## 4 DISCUSSION

This questionnaire survey aimed to investigate the needs and current circumstances of the “next generation” in HBP surgery, comprising the following four categories: (i) the board‐certification of JSHBPS, (ii) research activity and overseas study, (iii) recruiting, and (iv) work‐life balance. The majority of respondents seem to be “highly motivated”, “university‐affiliated” and “male” HBP surgeons, and their response highlighted the need for optimizing the HBP surgical training and supporting the research investigation. On the contrary, this survey showed HBP surgeons were not actively engaged in recruiting. Meanwhile, this survey demonstrated that HBP surgeons faced long working hours, and the vast majority recognized the need for work‐style reform.

The first section of the survey focused on the board certification of JSHBPS, which stands as the primary goal for every HBP surgeon and is highly prioritized, as presented in Figure 1. Before this certification system was launched by JSHBPS in 2011, there were no programs to monitor and ensure the quality of HBP surgeries.^4^, ^5^, ^6^, ^7^ The candidates for this certification are required to have operated more than 50 highly advanced HBP surgeries in the last 7 years, to submit detailed dictations and illustrations of all the surgeries, and to provide the recorded unedited video of one of the operated cases. The criteria for evaluation are strict, with a pass rate of approximately 50%, making it significantly more challenging than other board certifications, which typically have pass rates of around 80%. However, the most important issue was the system for the designation of board‐certified expert surgeons and safety management improved the mortality rate associated with highly advanced HBP surgeries.^4^, ^5^, ^8^ While the board certification system has reached a mature phase, several issues remain unresolved, as highlighted by various opinions. In addition, the operated casers for next generation HBP surgeons seemed to be insufficient. Balancing the demands of the next generation of HBP surgeons and the significance of the certification should be an ongoing topic of discussion.

The second section focused on research activity and overseas study, and primarily their activity seemed to be very high. As of the evidence, they have authored many papers as first authors throughout their career (supplementary document section 3.3–3.7—Data S1), and some surgeons experienced overseas study. Nevertheless, there was a disparity between the actual status and their willingness. This might be explained by the decreasing number of surgeons, limited financial support,^9^ or the isolated environment of Japanese institutions. Expanding the support for such surgeons and providing more information about overseas studies may allow for broadening the horizons of these highly motivated surgeons, leading to an increase in academic levels of Japanese HBP surgery.

The third section focused on the recruitment of new HBP surgeons. This study demonstrated that current HBP surgeons typically decide on their career path early in their surgical careers. It is noteworthy that nearly one‐third of respondents decided to pursue a career as an HBP surgeon either before choosing their medical specialty or during their time as medical students. Moreover, their motivation to become HBP surgeons is often driven by a desire to overcome the challenges associated with surgery. However, it should be noted whether this active trend can be consistent for future HBP surgeons. Although the situation would vary depending on the respective institutions, the activity of recruitment was not so evident (Figure 4d). Certainly, given the severe situation in the context of a decreasing number of surgeons, HBP surgeons who are under 45 years of age should have more weight regarding recruiting.

The final section focused on the work‐life balance. Although the number of staff surgeons varied across the institutions, the team‐based approach seemed to be practiced as reported by 60.1% of respondents. Nevertheless, 39.9% of respondents reported exceeding the work hours limit as stipulated by the new laws (i.e., 80 h). Notably, two‐thirds of respondents acknowledged the unsustainability of their current work styles. Burnout is a strong predictor of career dissatisfaction among surgeons,^10^ and the findings of this survey may serve as an important warning for the future HBP surgery. Besides, although the percentage of female gastrointestinal surgeons is increasing in Japan,^11^ gender equality remains an issue, with significant differences in the personal lives of surgeons based on gender and parental status.^12^ The traditional belief that women should bear primary family responsibilities persists among both male and female surgeons, possibly reflecting global trends of gender disparity.^12^, ^13^ Expanding the team‐based approach, offering incentives, task‐shifting from non‐surgical duties, and implementing structural improvements such as career sharing or on‐site child care could be potential solutions to these issues. However, these proposals are still challenging to implement due to the financial and time investments required from institutions and individuals.^14^, ^15^, ^16^ Dedicated, collective, and concerted efforts by both individuals and institutions where they work are crucial for achieving the most meaningful and impactful improvements in surgeon wellness.^17^

The results of this survey may help develop the fundamental policy of the NGP working group. How to minimize the gap between the current circumstances and the aspiration of next‐generation HBP surgeons represents a critical and urgent challenge. At the same time, it should be acknowledged that these surgeons are often faced with long working hours and insufficient training opportunities, which can lead to uncertainties about their future. Prioritizing the improvement of surgical experience and the reduction of work hours could sustain their surgical motivation, enhance work‐life balance, and create room for recruitment efforts. Challenges related to surgical education,^18^, ^19^, ^20^ research activity,^21^ work‐life balance,^12^, ^13^, ^14^, ^15^, ^16^ and recruitment^22^ appear to be similar across the globe and in other surgical fields. Sharing experiences and initiatives will be crucial in overcoming these challenges and ensuring the future sustainability of HBP surgery in Japan. The NGP working group can play a pivotal role in facilitating such opportunities.

The main limitation of this survey was the relatively low response rate of 17.5% and the composition of the study population, which consisted predominantly of male and university‐affiliated surgeons. As a result, the results might be biased and not fully reflect the perspectives of all young HBP surgeons. However, the ongoing centralization of HBP surgery in university hospitals and highly specialized centers in Japan suggests that this survey may provide valuable insights. Although the proportion of female HBP surgeons was not particularly low in this survey compared to those in JSHBPS members (approximately 4%, data from JSHBPS), the absolute number of female respondents was limited. Given the increasing number of female surgeons and the need to capture their perspectives, surveys that specifically focus on female surgeons may be required. Overall, future studies with a higher response rate and a more diverse population are needed to gain a more comprehensive understanding of the next generation of HBP surgeons in Japan.

## 5 CONCLUSIONS

This questionnaire survey highlighted the current status of the next generation HBP surgeons in Japan. Although they are motivated to acquire advanced surgical skills and recognize the importance of research experience, they are facing long working hours and insufficient training opportunities. Fundamental reforms, such as revising the training curriculum, improving work styles, and enhancing recruitment, are necessary steps forward. However, these are just the beginning; continued dialogue and collaboration are essential to develop comprehensive and sustainable reforms for the future of HBP surgery in Japan.

## AUTHOR CONTRIBUTIONS

YK‐F, TY, TH and KS: Study concept and design. All authors: Acquisition of data; analysis and interpretation of data. YK‐F, TY and TH: Drafting of the manuscript. All authors: Critical revision of the manuscript for important intellectual content. IE, MO, SE and KS: Study supervision.

## FUNDING INFORMATION

No financial support concerning this manuscript.

## CONFLICT OF INTEREST STATEMENT

The authors who have taken part in this study declare that they have nothing to disclose regarding funding or conflict of interest concerning this manuscript.

## PREVIOUS PRESENTATION

The content of this paper was presented in JSHBPS 2023.

## Supporting information


**Data S1:** Supporting Information.

## Data Availability

The data supporting this study's findings are available from the corresponding author.
