# Supplementary material for: It is time for reform: Results from a questionnaire survey on the current status of next generation HBP surgeons in Japan
Source: J Hepatobiliary Pancreat Sci. 2024 Dec 10;32(1):17–25. doi: 10.1002/jhbp.12092 (PMC11780308; doi:10.1002/jhbp.12092)
Supplement: Supplementary file 1 — Data S1: Supporting Information. [file JHBP-32-17-s001.docx]

SUPPLEMENTARY FILE

Title:

It is Time for Reform: Results from a Questionnaire Survey on the Current Status of Next Generation HBP Surgeons in Japan.

**AUTHORS**

Yukiko Kosai-Fujimoto^1), 2), 3)^, Tomoaki Yoh^1), 4)^, Takanobu Hara^1), 5)^, Saori Umezawa^1), 6)^, Aya Maekawa^1), 7), 8)^, Yasuko Matsuo^1), 9)^, Norihiro Ishii^1), 10), 11)^, Hiroko Okinaga^1), 12)^, Itaru Endo^13), 14)^, Masayuki Ohtsuka^13), 15)^, Susumu Eguchi ^5),13)^, Ken Shirabe^10), 13)^ *.

**AFFILIATIONS:**

1. Next Generation Project Working Group in Japanese Society of Hepato-Biliary-Pancreatic Surgery, Tokyo, Japan

2. Department of Surgery, NHO Fukuokahigashi Medical Center, Fukuoka, Japan.

3. Department of Surgery and Science, Graduate School of Medical Sciences, Kyushu University, Fukuoka, Japan.

4. Department of Surgery, Graduate School of Medicine, Kyoto University, Kyoto, Japan.

5. Department of Surgery, Nagasaki University Graduate School of Biomedical Sciences, Nagasaki, Japan.

6. Department of Gastroenterological and General Surgery, St. Marianna University School of Medicine, Kanagawa, Japan.

7. Division of Hepatobiliary and Pancreatic Surgery, Cancer Institute, Hospital, Japanese Foundation for Cancer Research, Tokyo, Japan.

8. Department of Hepatobiliary and Pancreatic Surgery, Graduate School of Medicine, Tokyo Medical and Dental University, Tokyo, Japan

9. Department of Surgery, Nara Medical University, Nara, Japan

10. Division of Hepatobiliary and Pancreatic Surgery, Department of General Surgical Science, Graduate School of Medicine, Gunma University, Gunma, Japan.

11. Department of Surgery, Gunma Saiseikai Maebashi Hospital, Maebashi, Gunma, Japan.

12. Department of Hepato-Biliary-Pancreatic Surgery, Tokyo Metropolitan Cancer and Infectious Diseases Center Komagome Hospital, Tokyo, Japan.

13. Japanese Society of Hepato-Biliary-Pancreatic Surgery, Tokyo, Japan

14. Department of Gastroenterological Surgery, Yokohama City University School of Medicine, Graduate School of Medicine, Yokohama, Japan.

15. Department of General Surgery, Chiba University Graduate School of Medicine, Chiba, Japan.

***CORRESPONDING AUTHOR:**

Ken SHIRABE

Division of Hepatobiliary and Pancreatic Surgery, Department of General Surgery, Graduate School of Medicine, Gunma University, 3-39-22, Showamachi, Maebashi, 371-8511, Japan.

Tel: +81-27-220-8224

Fax: +81-27-220-8230

E-mail: kshirabe@gunma-u.ac.jp

**SUPPLEMENTARY FIGURES**

**SUPPLEMENTARY FIGURE 1**

The doctors’ work style reform in Japan

**SUPPLEMENTARY FIGURE 2**

The JSHBPS Board Certification System

**SUPPLEMENTARY DOCUMENT**: Questions in the Survey

**SECTION 1**. Background Characteristics of the Answerers

- 1. Age. **(TABLE 1)**
  2. Postgraduate year as a doctor **(TABLE 1)**
  3. Gender **(TABLE 1)**
  4. The current position at work (**SUPPLEMENTARY FIGURE BELOW**)
  5. The type of the hospital of your current employment **(TABLE 1)**
  6. The affiliation/non-affiliation to the university surgery departments **(TABLE 1)**
  7. The licenses/certifications you have already obtained **(TABLE 1)**
  8. The licenses/certifications of your priority **(TABLE 1)**

**SECTION 2.** The Board Certification System of JSHBPS.

2.1 Do you work at the board-certified hospitals of JSHBPS? (**FIGURE 1B**)

2.2 How long have you worked in the board-certified hospitals after PGY3? (**FIGURE 1C**)

2.3 How many highly advanced HBP surgeries have you operated in your lifetime? (**FIGURE 1D**)

2.4 How many highly advanced HBP surgeries have you operated on in 2021? (**FIGURE 1E**)

2.5 How many HBP surgeries do you perform on average in a month except for laparoscopic cholecystectomy? (**SUPPLEMENTARY FIGURE BELOW**)

2.6 Please write down your opinions about the board certification system. (**SUPPLEMENTARY TABLE BELOW**)

| The requests to the JSHBPS about the board certification system |
| --- |
| ・Establishment of financial incentives |
| ・Official airing of the significance of board-certified HBP surgeons |
| ・Clarification and transparency of judgement criteria |
| ・Take measures against the negative effects of the board certification system on the training for young HBP surgeon |
| ・Workshops and seminars for candidate surgeons |
| ・Release of the ideal videos on the society website |
| ・Approval of cases at non-certified hospitals  ・Decrease of the number of surgeries required for certification |
| *The similar answers are summarized. |

**SECTION 3**. Research Activity and Overseas Study.

3.1 Have you ever done any basic or clinical research? (**FIGURE 2A**)

3.2 Do you think the research activity is necessary for your career formation? (**FIGURE 2B**)

3.3 How many scientific papers have you written in Japanese or English? (**SUPPLEMENTARY FIGURE BELOW**)

3.4 How many scientific papers have you written? (Original articles only)

(**SUPPLEMENTARY FIGURE BELOW**)

3.5 How many scientific papers have you written? (Case reports)

(**SUPPLEMENTARY FIGURE BELOW**)

3.6 How many scientific papers have you written? (Letters, reviews, etc.)

(**SUPPLEMENTARY FIGURE BELOW**)

3.7 How many scientific papers have you written? (English articles of any kind)

(**SUPPLEMENTARY FIGURE BELOW**)

3.8 Would you apply for the JSHBPS Project Study if there is a project only for younger surgeons? (**SUPPLEMENTARY FIGURE BELOW**)

3.9 Have you ever studied abroad or in domestic institutions other than your affiliation as a doctor? (**FIGURE 2C**)

3.10 Are you willing to study abroad or in domestic institutions other than your affiliation as a doctor in the future? (**FIGURE 2D**)

3.11 Are you interested in the Kenneth Warren Fellowship Program sponsored by IHPBA?

(**SUPPLEMENTARY FIGURE BELOW**)

**SECTION 4**. Recruiting

4.1 When did you decide to become an HBP surgeon? (**FIGURE 3A**)

4.2 What are the reasons you became an HBP surgeon? (**FIGURE 3B**)

4.3 Is there any website or social media platform for deciding the future plan used by you or colleagues around you as an HBP surgeon?

(**SUPPLEMENTARY FIGURE BELOW**)

4.4 What do you think is necessary for recruiting young HBP surgeons?

(**SUPPLEMENTARY FIGURE BELOW**)


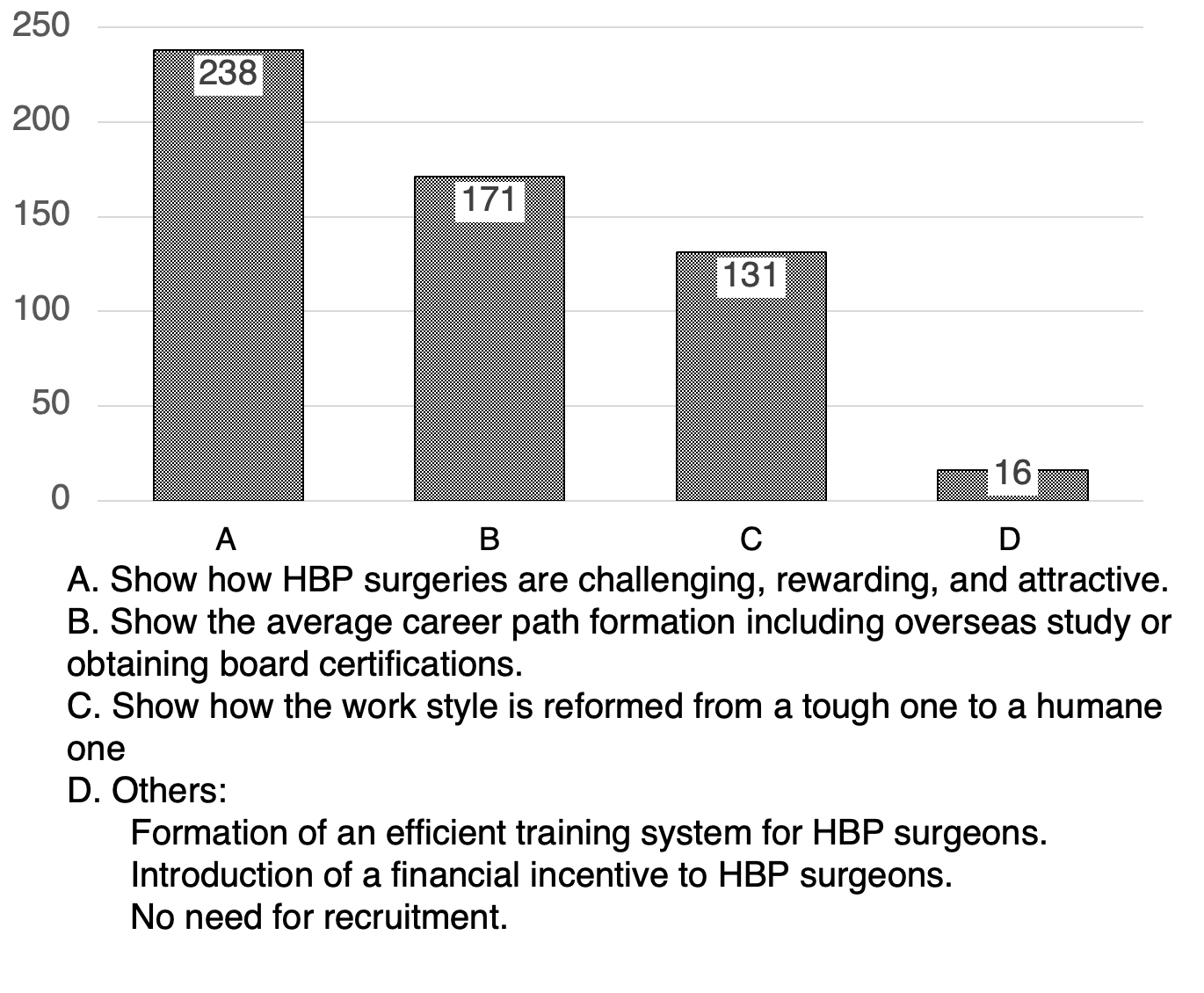


4.5 How many young HBP surgeons come to the HBP surgery department of your institution? (**FIGURE 3C**)

4.6 Do you do any recruiting activity as the HBP surgery department of your institution? (**FIGURE 3D**)

Section 5. Work-life balance.

5.1 Do you belong to the HBP-specialized department or the general surgery department?

(**SUPPLEMENTARY FIGURE BELOW**)

5.2 How many HBP surgeons belong to your hospital? (**FIGURE 4A**)

5.3 How many female HBP surgeons work in your hospital?

(**SUPPLEMENTARY FIGURE BELOW**)

5.4 Do you work in a team-based system or a doctor-based system? (**FIGURE 4B**)

5.5 What do you think about the team-based work style?

(**SUPPLEMENTARY FIGURE BELOW**)

5.6 How many complete day-offs do you have in a month? (**FIGURE 4C**)

5.7 How many days of paid leave do you have in a year?

(**SUPPLEMENTARY FIGURE BELOW**)

5.8 How many times per month do you work the night shift?

(**SUPPLEMENTARY FIGURE BELOW**)

5.9 How long do you work after your duty hours in a month? (**FIGURE 4D**)

5.10 What type of work is demanded in your hospital after the night shift? (**FIGURE 4E**)

5.11 Do you have a partner in your private life? (**SUPPLEMENTARY FIGURE BELOW**)

5.12 Does your partner have a job? (**SUPPLEMENTARY FIGURE BELOW**)

5.13 How do you share the household chores with your partner?

(**SUPPLEMENTARY FIGURE BELOW**)

5.14 Is it possible for you to join the events of your family members such as the entrance ceremony of the kids’ school? (**SUPPLEMENTARY FIGURE BELOW**)

5.15 Do you think HBP surgeons must promote the improvement of work-life balance?

(**SUPPLEMENTARY FIGURE BELOW**)

5.16 What do you think is necessary to improve the working environment of HBP surgeons?

(**SUPPLEMENTARY FIGURE BELOW**)

5.17 Which measure do you think is feasible in the HBP surgery department?

(**SUPPLEMENTARY FIGURE BELOW**)

5.18 Can you maintain your current work style a decade later? (**FIGURE 4F**)

5.19 What do you want the JSHBPS to do regarding work-life balance improvement?

(**SUPPLEMENTARY TABLE BELOW**)

| ・Introduction of interval between work shifts |
| --- |
| ・Task shifting to non-MD staffs for reducing the overwork |
| ・Improvement of salary |
| ・Promotion of team-based work style and on-call systems |
| ・Career support for postpartum female surgeons |
| ・Promotion of financial incentives for board-certificated surgeons |
| *The similar answers are summarized. |
